# Oil-in-Water Emulsions Stabilized by Cellulose Nanofibrils—The Effects of Ionic Strength and pH

**DOI:** 10.3390/nano9020259

**Published:** 2019-02-14

**Authors:** Ragnhild Aaen, Fredrik Wernersson Brodin, Sébastien Simon, Ellinor Bævre Heggset, Kristin Syverud

**Affiliations:** 1 Ugelstad Laboratory, Department of Chemical Engineering, Norwegian University of Science and Technology, N-7491 Trondheim, Norway; ragnhild.aaen@ntnu.no (R.A.); sebastien.c.simon@ntnu.no (S.S.); 2 RISE PFI, N-7491 Trondheim, Norway; Fredrik.brodin@rise-pfi.no (F.W.B.); ellinor.heggset@rise-pfi.no (E.B.H.)

**Keywords:** cellulose nanofibrils (CNFs), o/w emulsions, emulsion stability, TEMPO-oxidation, enzymatical treatment, nanocelluloses

## Abstract

Pickering o/w emulsions prepared with 40 wt % rapeseed oil were stabilized with the use of low charged enzymatically treated cellulose nanofibrils (CNFs) and highly charged 2,2,6,6-tetramethylpiperidine-1-oxyl (TEMPO)-oxidized CNFs. The emulsion-forming abilities and storage stability of the two qualities were tested in the presence of NaCl and acetic acid, at concentrations relevant to food applications. Food emulsions may be an important future application area for CNFs due to their availability and excellent viscosifying abilities. The emulsion characterization was carried out by visual inspection, light microscopy, viscosity measurements, dynamic light scattering and mild centrifugation, which showed that stable emulsions could be obtained for both CNF qualities in the absence of salt and acid. In addition, the enzymatically stabilized CNFs were able to stabilize emulsions in the presence of acid and NaCl, with little change in the appearance or droplet size distribution over one month of storage at room temperature. The work showed that enzymatically treated CNFs could be suitable for use in food systems where NaCl and acid are present, while the more highly charged TEMPO-CNFs might be more suited for other applications, where they can contribute to a high emulsion viscosity even at low concentrations.

## 1. Introduction

Emulsions, systems where droplets of one immiscible liquid are dispersed in another liquid, are important in many different applications, ranging from oil recovery, via cosmetics and pharmaceuticals, to food applications. Emulsions are thermodynamically unstable, and emulsifiers or stabilizers are required to keep the emulsions kinetically stable for a longer period. Surfactants are effective for this purpose, but the release of surfactants into the aquatic and terrestrial environment through wastewater poses a major pollution problem [1,2,3]. Another way of stabilizing emulsions is with solid particles, giving rise to what is known as Pickering emulsions [4,5]. The particles can irreversibly adsorb at liquid interfaces, forming a packed layer at the interface, thus providing steric stabilization towards coalescence [6,7,8]. In addition, the particles can form a network in the continuous phase, impeding the coalescence of the droplets, and increasing the viscosity, slowing down the rate of creaming or sedimentation [9,10,11]. The wettability of the particles is important to decide what kind of emulsion they can stabilize. Particles with a more hydrophilic character can stabilize o/w emulsions, while the more hydrophobic particles can stabilize w/o emulsions [6,12].

Cellulose nanofibrils (CNFs) are a renewable and abundant resource, and due to their amphiphilic properties, they can be used for stabilization in Pickering emulsions [12,13,14]. CNFs with different charge densities and morphologies have been used to stabilize o/w emulsions. These studies ranges from highly charged finely fibrillated TEMPO-oxidized CNFs to less charged bacterial cellulose, enzymatically treated, and to coarser, purely mechanically disintegrated CNFs [13,14,15,16,17,18,19,20]. Saelices and Capron have compared the features of emulsions stabilized by four types of nanocellulose samples [21]. They successfully report the production of nanosized droplets for CNC and TEMPO oxidized CNF using HP-homogenizer. However, the less fibrillated CNF would only result in the production of emulsions with average diameter of 5 μm. The emulsions produced with CNFs were generally stable towards coalescence during storage up to several months [15,17,20]. The mechanisms for this stability seems to be a combination of adsorption of fibrils to the oil-water interface, and the establishment of a gel-like viscoelastic network of fibrils, posing a mechanical barrier towards coalescence of the oil droplets [14,17,18,20]. Some groups have reported that the emulsions stabilized by CNFs were susceptible to creaming, especially at lower CNF concentrations [13,20]. Creaming is a phenomenon in which the droplets migrate and accumulate at the top of the container due to the density differences between the dispersed and the continuous phase. Subjecting CNF-stabilized emulsions to mild centrifugation have resulted in creaming and release of free water by emulsions stabilized by enzymatically treated CNFs [18]. Gestranius et al. [13] reported that centrifugation of emulsions stabilized with mechanically fibrillated CNFs or TEMPO-oxidized CNFs, led to denser packing of the oil droplets in the already existing creaming layer, and there was some free oil at the top of the emulsions, indicating coalescence of some of the oil droplets. Generally, a decrease in the average droplet size of the emulsion can be expected as the CNF concentration increases, due to a better surface coverage of the droplets, as well as an increase in viscosity of the continuous phase [16,17,20]. However, at high enough CNF concentrations, the high viscosity can lead to poorer homogenization of the emulsion, resulting in broader droplet size distributions and larger average droplet sizes for the emulsions [13,20].

Cellulose nanocrystals (CNCs), which are shorter than CNFs, have also been used by several groups to stabilize o/w emulsions [22,23,24,25]. The main stabilization mechanism for these CNCs is adsorption at the oil-water interface, with a denser coverage of the droplets for shorter nanocrystals [23,24]. Comparing emulsions stabilized by CNFs and CNCs, the CNC stabilization allows for the formation of smaller droplets, as the shorter CNCs can accommodate a larger curvature of the oil droplets [12,16,23]. However, these emulsions may be more vulnerable to coalescence, high shear and temperature, as they do not have the same ability as CNFs to form strong networks in the continuous phase [13]. Surface-modified CNFs and CNCs have also been shown to stabilize w/o emulsions, as the surface of cellulosic materials are readily available for surface modifications such as silylation or esterification, making the fibrils more hydrophobic [11,12,26,27,28].

One of the possible applications for CNF-stabilized emulsions is within the food industry. Use of CNFs within food systems does, however, require some tolerance towards other food ingredients, such as NaCl or mild acids. Due to the negative charge of CNFs, their rheological properties may change as the ionic strength or pH changes, especially for highly charged fibrils such as TEMPO-oxidized or carboxymethylated CNFs [29,30,31,32]. Despite this, it has been shown that emulsions stabilized by bacterial cellulose have proved stable towards changes in ionic strength, temperature and pH [14,17].

The aim of this work has been to compare the ability of low charged enzymatically treated CNFs and highly charged TEMPO-oxidized CNFs to stabilize o/w emulsions with 40 wt % rapeseed oil, in a model system with a similar fat content to low-fat mayonnaise. The emulsion-forming abilities of the two qualities were tested in the presence of NaCl and acetic acid, at concentrations relevant to food applications. The emulsion characterization was carried out by ocular inspection, light microscopy, viscosity measurements, dynamic light scattering and mild centrifugation. Stable emulsions in the absence of salt and acid were obtained for both CNF qualities, and the enzymatically stabilized CNFs were able to stabilize emulsions in the presence of acid and NaCl.

## 2. Materials and Methods

### 2.1. Materials

An industrially produced, never-dried, totally chlorine free (TCF)-bleached sulfite dissolving pulp obtained from Domsjö Fabriker (Domsjö Mill, Domsjö, Sweden) was used as raw material for production of the two CNF grades. The pulp was produced from a mixture of 60% spruce (*Picea abies*) and 40% pine (*Pinus sylvestris*). All other chemicals were of laboratory grade quality and purchased from Sigma Aldrich (St. Louis, MO, USA).

#### 2.1.1. Enzymatically Pretreated CNFs (CNF-E)

A sample of enzymatically pretreated CNFs was provided by Innventia. The materials used and the preparation procedure have been described elsewhere [33]. The pretreatment started with a refining step to make the fiber wall more accessible to the endoglucanase enzymes. After a 2 h enzyme treatment conducted at neutral pH, the pulp was treated in a second refining step, followed by a washing step and dilution to 2% consistency. After dilution, the pretreated pulp was fibrillated by passing the pulp five times through a Microfluidizer (M-110EH, Microfluidics Corp. Westwood, MA, USA) at 1700 bar pressure. The microfluidizer had two z-shaped interaction chambers (200 μm + 100 μm).

#### 2.1.2. TEMPO-Oxidized CNFs (CNF-T)

TEMPO-oxidized CNFs were prepared from Domsjö dissolving pulp based on the procedure described by Saito et al. [34]. One hundred grams (dry weight) of pulp were oxidized at 1.33% consistency in a reaction system containing 1.25 g of TEMPO and 12.5 g of NaBr. The dosage level of NaClO was 3.8 mmol/g pulp and the reaction was conducted at room temperature (~20 °C). The pH of the mixture was maintained at 10 by gradual addition of 0.5 M NaOH. The reaction was ended when the no further decrease in pH was observed. At that point, the pH was adjusted to 7 by addition of 0.5 M HCl. The oxidized pulp was washed with de-ionized water using a Buchner funnel until the conductivity of the filtrate was less than 5 μS/cm. The pulp was stored at 4 °C prior to fibrillation. After dilution to 2% pulp consistency, the TEMPO-oxidized pulp was passed once through the Microfluidizer (M-110EH, Microfluidics Corp. Westwood, MA, USA). The same pressure level and interaction chambers were used as for the enzymatically pre-treated pulp.

### 2.2. Characterization of Rapeseed Oil and CNFs

#### 2.2.1. Characterization of Rapeseed Oil

The rapeseed oil was subjected to density measurements in duplicate in a density meter (DMA 5000, Anton Paar GmbH, Graz, Austria), with measurements performed from 15 to 60 °C. The interfacial tension (IFT) between the rapeseed oil and water was measured in duplicate with a Sigma 70 tensiometer (KSV Instruments Ltd., Helsinki, Finland) equipped with a Platinum Du Noüy ring. The experiment was conducted at 20 °C, and no equilibrium state was reached during the 23 h the IFT values were recorded. The reported value is the average of two measurements, recorded after 23 h. The refractive index of the oil was determined with an ARAGO refractometer (Cordouan Technologies, Pessac, France). The experiment was performed in triplicate, with 20 measurements in each. The refractive index of the rapeseed oil was calculated as the average value for all the 60 measurements.

#### 2.2.2. Characterization of CNF Film

Films of 10 g/m^2^ were prepared in petri dishes by solvent casting of the nanocellulose dispersions. The surfaces of CNF-films made from the two qualities were characterized by scanning electron microscopy (SEM) (Hitachi S-3000N, Hitachi Scientific Instruments, Tokyo, Japan). Images were recorded at 100× and 10,000× magnification using a secondary electron detector. The fraction of residual fibers that were not fibrillated during microfluidization was determined by a method developed by Chinga-Carrasco et al. [35]. The CNF suspensions were diluted to 0.25% consistency before analysis, and a L&W Fiber tester plus was used to characterize fiber dimensions and number of fibers present in the two CNF samples. The total amount of charged groups in the pulps was determined by conductometric titration, the enzymatically treated quality according to SCAN-CM65:02, and the TEMPO-oxidized pulp according to the procedure described by Saito and Isogai [36].

### 2.3. Preparation of O/W Model Emulsions

Emulsions were prepared by using a two-step procedure with pre-mixing followed by homogenization. Samples were first pre-emulsified using a Silverson L4R high-shear mixing device at 4000 rpm for 3 min to obtain coarse emulsions before homogenization. After pre-mixing, the sample was passed once through a two-stage homogenizer (APV Gaulin LAB 60/500/2, APV-Schröder, Lübeck, Germany) with a total pressure drop of 200 bars, and with the second-stage pressure set to 30 bars. This two-stage homogenization procedure with 10–15% back-pressure is known to be ideal for preparing o/w emulsions where the second-stage valve intensifies turbulence and prevents two-phase flow. The final emulsions were collected in 10 L plastic buckets. Subsequently, small sub-samples were collected from the outlet pipe of the homogenizer to be used in assessment of visual stability, droplet size distribution and light microscopy. All samples were stored in a climate room at 23 ± 1 °C. The homogenizer was cleaned extensively between each run, and the first 550 mL of liquid from the homogenizer were discarded to avoid dilution effects.

The emulsions were based on a mixture of rapeseed oil and water. All samples contained 1 kg rapeseed oil (40 wt %), and deionized water was added so that the total weight of each sample was 2.5 kg. A series of eight emulsion samples were prepared containing 0.5 wt % CNFs, in addition to NaCl (1.0 wt %) and acetic acid (0.2 wt %); see Table 1. The content of NaCl and acetic acid are set to be representative for a food emulsion, such as mayonnaise [37].

### 2.4. Characterization of O/W Emulsions

The visual appearance of the emulsions was assessed by visual inspection and by recording photos with a digital camera. Visual stability was evaluated one day, seven days and 28 days after emulsion preparation. The emulsions were stored in 15-mL plastic tubes placed vertically in a stand. Samples for microcopy analysis were prepared by placing a small droplet of emulsion sample between a microscopy glass slide and a cover glass.

Light microscopy (Leitz DM RXE, Leica Microsystems, Wetzlar, Germany) images were recorded at 400× magnification of a thin layer of emulsion to obtain qualitative information about emulsion homogeneity and droplet sizes.

The pH was measured for both CNF dispersions alone, and for the emulsions.

The size distributions of oil droplets were determined right after preparation, after one day, one week and one month by laser light diffraction in a Mastersizer 3000 (Malvern Panalytical, Worcester, UK). The sample tube was turned gently upside down before the sample was taken out for analysis, and the measurements were run with eight replicates for each sample. Histograms of size distribution are given in volume % vs. droplet diameter.

The apparent viscosity of the oil and the emulsions was measured in duplicate with a Brookfield DV2T-RV viscometer equipped with V-71 or V72 spindle (Brookfield Engineering Laboratories, Inc., Middleboro, MA, USA). Five hundred milliliters of sample were poured into a 600-mL glass beaker. The sample was analyzed for 5 min at 10 rpm. The apparent viscosity is reported as the end-point value.

The emulsions were subjected to centrifugation on the day after preparation, in an accelerated stability test (Labofuge 400 R, Heraeus Instruments, Hanau, Germany). Two replicates for each sample were subjected to a relative centrifugal force (RCF) of 2958 × g for 5 min at 23 °C, and the emulsions were photographed before and after the centrifugation. The emulsions were inspected visually for creaming and phase separation.

## 3. Results and Discussion

### 3.1. Characterization of CNF Samples

The introduction of charges by TEMPO-mediated oxidation led to large differences between the pulps regarding the content of carboxyl groups after pretreatment, as shown in Table 2. The much higher charge density of CNF-T compared to CNF-E may have large impact on the ability and function as a stabilizing agent in o/w emulsions. The differences in morphology between CNF-E and CNF-T may also influence the ability to stabilize o/w emulsions. The residual fiber data in Table 2 show that the fibrillation treatment resulted in CNF samples with low residual fiber content. This means that the CNF samples consist mainly of fibrillated material, which have shorter lengths and narrower widths than residual fibers. The CNF-T sample had a higher proportion of residual fiber than the CNF-E sample, but it should be noted that this sample had been treated fewer passes through the microfluidizer. Generally, a low residual fiber content is beneficial in many food applications since large particles can be sensed, and affect mouthfeel in a negative way.

**Figure 1:**
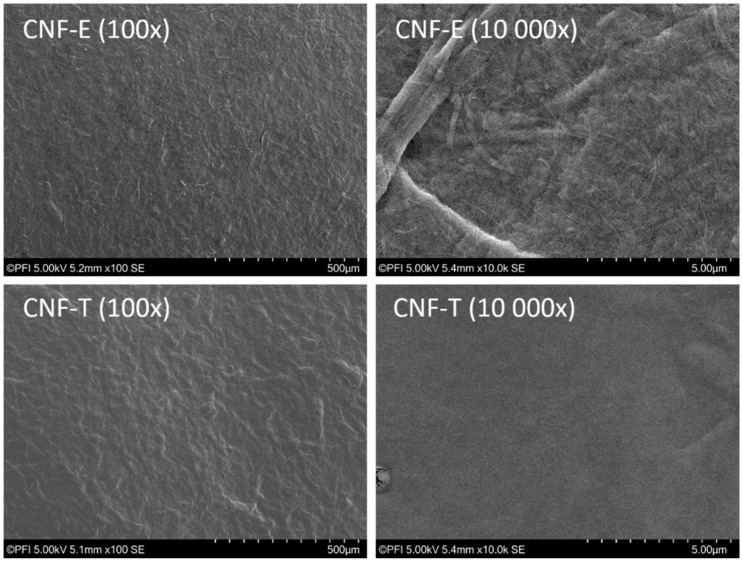
Scanning electron microscopy (SEM) images of the top surface of air-dried CNF films recorded at 100× and 10,000× magnification of enzymatically treated CNFs (upper) and TEMPO-oxidized CNFs (lower).

In Figure 1, scanning electron microscopy images of the CNF films are shown to illustrate the differences in morphology between the two CNF samples. At low magnification level (100×), it is seen that the CNF-T sample contains a higher amount and larger size of residual fibers than the CNF-E sample. In both samples, the residual fibers are embedded in a dense and continuous matrix of CNFs. At high magnification level (10,000×), it is seen that the CNF-E sample consists of both narrow nanofibril particles but also a significant portion of larger fibrillar aggregates. Pääkkö et al. [38] have reported that enzymatically treated CNFs results in a mixture of mainly narrow nanofibrils having widths of about 5 nm and fibril aggregates with widths of about 10–20 nm. The CNF-T film has a much smoother surface and the individual CNFs are, as expected, too narrow to be observed at this level of magnification. TEMPO-oxidized CNFs generally have widths of 3–4 nm [34].

### 3.2. Characterization of the Rapeseed Oil

The refractive index for rapeseed oil was measured with a refractometer to be 1.46882 ± 0.00006, while the density of the oil was determined to be 0.91663 ± 0.00003 g/cm^3^ at 20 °C, with the uncertainty given as standard deviation and max value–min value respectively. The interfacial tension was measured to 10.9 ± 0.3 mN/m, which is a rather low value, indicating the presence of some natural surface-active components in the oil, probably the fatty acids. These will adsorb onto the oil droplets and influence the emulsion stability.

### 3.3. The Effect of Addition of Salt and Acid on CNF-Stabilized Emulsions

#### 3.3.1. Apparent Viscosity and pH

The apparent viscosity of rapeseed oil was determined to be 64.2 ± 1.3 mPa∙s. The apparent viscosity for the CNFstabilized emulsions containing acetic acid or NaCl, is shown in Table 3, while the pH values are shown in Table 4.

The CNF-T-stabilized emulsion suffers a large loss of viscosity upon the addition of acetic acid and NaCl, indicating aggregation and collapse of the fibril network formed in the continuous phase. This is not a surprising finding, as the fibril network is stabilized in part by the strong electrostatic repulsion between the fibrils [34]. Addition of NaCl causes screening of the negative charges on the fibril surface, resulting in less repulsion between the oil droplets where CNF-T is adsorbed on the surface [39]. While the screening of charges can lead to closer fibril-fibril interactions and a stronger network, it can also, especially under shear, lead to irreversible fibril aggregation, giving a lower bulk viscosity [29,31,32,40]. This aggregation of fibrils can in turn reduce the emulsion stability both by reducing the viscosity of the medium, and, as shown for charged CNCs, lead to desorption from the oil-water interface [32,39]. Lowering the pH by addition of acetic acid will lead to protonation of some of the surface carboxyl groups, thus lowering the negative charge of the fibril surface, and give similar effects to those observed for increased ionic strength [41]. At the same time, stronger hydrogen bonding between fibrils can be achieved at low pH where the carboxylate groups have been protonated, and several research groups have produced strong CNF hydrogels by lowering the pH [30,40,42]. As given in Table 4, the pH for the CNF-T emulsion with added acid is 3.95 for acid alone, and 3.42 for acid and NaCl, which is in the same range as the pKa value (3–4) found for the C6-carboxyl groups [42,43]. The protonation of approximately half of the carboxyl groups leads to a destabilization of the fibril network as the charge density is reduced. At the same time, protonation also allows for stronger hydrogen bonding between fibrils, which may counter the destabilization to some extent. Indeed, for the CNF-E-stabilized emulsion, which is not stabilized by electrostatic repulsions to the same degree as the CNF-T-stabilized emulsion, the lowering of pH from 7.52 to 3.22 led to a higher viscosity than for the reference emulsion.

Wågberg et al. [44] found that higher ionic strength affected the dissociation behavior of carboxyl groups on CNFs by increasing the degree of dissociation. This is supported by our results, as the pH is lower for the emulsions where both acid and NaCl is present, compared to acid alone. The viscosity values for these emulsions are somewhere in between the values obtained for the emulsions with only one of the components present. This may indicate some balance between the destabilizing effects of adding NaCl, and the partly stabilizing effects of lowering the pH.

#### 3.3.2. Visual Stability

**Figure 2:**
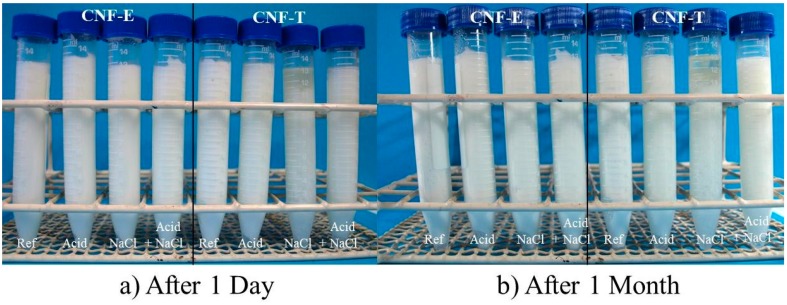
Pictures of the CNF-stabilized emulsions with 0.5 wt % CNFs, containing acetic acid and/or salt. The pictures are taken after (**a**) one day and (**b**) one month of storage at 23 °C.

The visual stability of the emulsions were evaluated by taking photographs of the samples at one day, one week and one month after the emulsification. The pictures taken of the samples after one day and one month of storage at 23 °C are shown in Figure 2.

The CNF-T-stabilized emulsion containing NaCl has a clear oil layer on top even right after preparation, while this is not observed for any of the other emulsions. The clear areas observed in the bottom of several of the tubes may be some free water due to the creaming of the droplets, but can also be trapped air bubbles.

#### 3.3.3. Light Microscopy Images and Droplet Size Distribution

**Figure 3:**
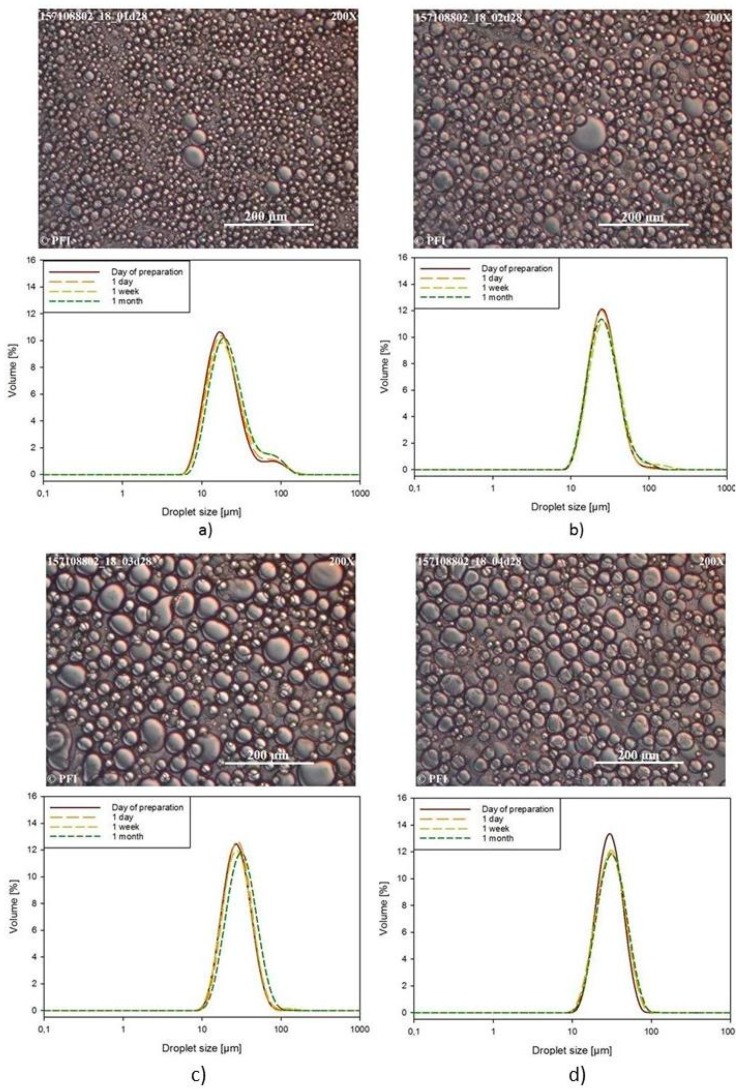
Light microscopy images taken after one month of storage, as well as the droplet size distributions over time for emulsions stabilized with 0.5 wt % CNF-E, for (**a**) a reference emulsion, (**b**) an emulsion with 0.2 wt % acetic acid, (**c**) an emulsion with 1.0 wt % NaCl and (**d**) an emulsion with 0.2 wt % acetic acid and 1.0 wt % NaCl.

**Figure 4:**
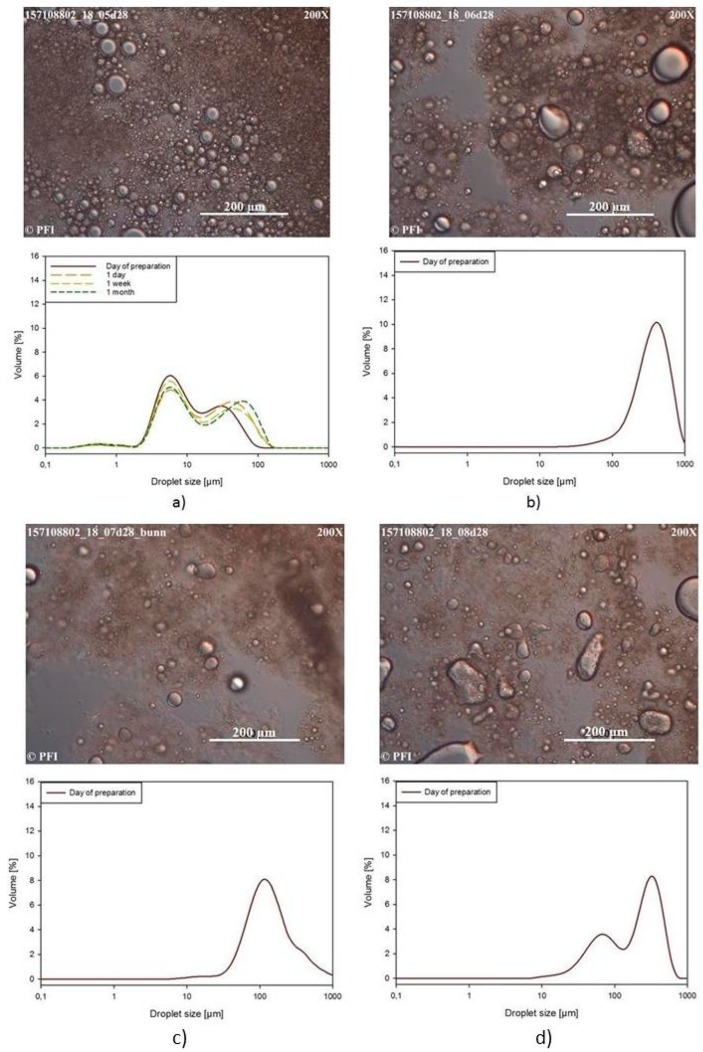
Light microscopy images taken after one month of storage, as well as the droplet size distributions over time for emulsions stabilized with 0.5 wt % CNF-T, for (**a**) a reference emulsion, (**b**) an emulsion with 0.2 wt % acetic acid, (**c**) an emulsion with 1.0 wt % NaCl and (**d**) an emulsion with 0.2 wt % acetic acid and 1.0 wt % NaCl.

The morphology and droplet size distribution of the emulsions with added acid or NaCl, were followed from the day of preparation, after one day, one week and one month. The light microscopy images taken after one month of storage at 23 °C, together with the droplet size distribution of the emulsions followed over one month are shown in Figure 3 and Figure 4. Figure 3 shows the results for the CNF-E-stabilized emulsions, while Figure 4 shows the results for the CNF-T-stabilized emulsions.

Both the microscope pictures and the droplet size distributions show that all the samples are polydisperse, with droplets in the range from 10 µm and up to almost 100 µm. The mastersizer is not able to distinguish between individual droplets and aggregates of smaller droplets. Consequently, some of the sizes measured could correspond to this type of aggregates as previously has been shown with TEMPO-CNF-stabilized emulsions [21]. Low charge CNFs, such as bacterial cellulose, have been shown to give emulsions that are stable against changes in pH and ionic strength [14,17]. With addition of acetic acid or NaCl to the emulsions stabilized by 0.5 wt % CNF-E (Figure 3), the average droplet size of the emulsion increases, as indicated by both the micrographs and the droplet size distributions. There is an increase in the volume average diameter (d_3;4_) from 25.4 ± 0.9 µm for the reference sample, to 30.3 ± 0.8 µm when acid is added, 31.5 ± 0.7 µm with added NaCl, and 33.5 ± 1.0 µm for the emulsion containing both acid and NaCl. However, all the emulsions are fairly time stable, with only minor changes in the droplet size distributions over the one month of storage.

The addition of acetic acid or NaCl to CNF-T-stabilized emulsions (Figure 4), led to the formation of highly unstable emulsions, with large initial droplet sizes and rapid coalescence. As mentioned before, the change of pH or ionic strength increases the likelihood of aggregation of the highly charged fibrils, causing a less structured water phase and less repulsion between oil droplets, which can both contribute to reduced emulsion stability. CNFs prepared by TEMPO-oxidation are clearly not suitable to stabilize emulsions where NaCl or acid is present in the concentrations used here, while the enzymatically treated CNFs are able to stabilize emulsion at low pH and high ionic strength.

#### 3.3.4. Accelerated Stability Test

**Figure 5:**
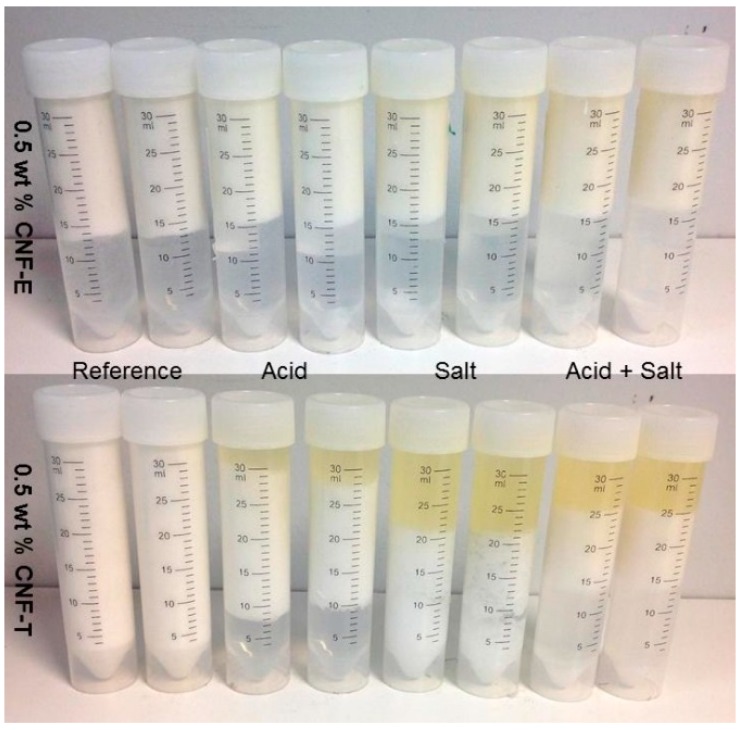
Pictures of emulsions stabilized by CNF-E (upper) or CNF-T (lower) with the addition of acetic acid and NaCl, taken after centrifugation, with two replicates for each sample.

The emulsions were subjected to centrifugation to compare the different types of CNFs and the effect of addition of NaCl or acetic acid, with results shown in Figure 5.

The CNF-E-stabilized emulsions all show the same behavior, with release of free water, but no oil after centrifugation. This is in agreement with the results obtained by Ström et al., who found that emulsions stabilized by enzymatically treated CNFs formed a free water phase after centrifugation [18]. This result suggests that the CNFs, in CNF-E-stabilized emulsions, form a relatively stable network in the continuous water phase, entrapping the oil droplets as well as adsorbing at the o/w interface. The emulsion stabilized with 0.5 wt % CNF-T showed no observable changes after centrifugation, and was thus the most stable one in the accelerated stability test. This is likely an effect of the very high viscosity obtained by this specific emulsion: 38300 ± 200 mPa·s compared to 5300 ± 300 mPa·s for CNF-E. The CNF-T-stabilized emulsions containing acetic acid or NaCl, on the other hand, all suffers from destabilization through coalescence, leaving a distinct oil layer on top of the container, as well as a free water phase at the bottom. The largest release of oil can be seen for the emulsion containing NaCl, which was also the emulsion with the lowest measured viscosity.

The viscosity seems to be a very important factor for the stability of CNF-stabilized emulsions, where high viscosity leads to more stable emulsions. The charge density of the CNFs plays a large role in the stability of emulsions subjected to changes in ionic strength or pH, and for applications within food industry the enzymatically treated CNFs appears to be a better choice than the CNFs subjected to TEMPO-mediated oxidation. Further work could include studies on the performance of enzymatically treated CNFs in actual food systems, as well as work towards gaining a deeper understanding of the food safety of using CNFs in food.

## 4. Conclusions

Pickering emulsions (o/w) with 40 wt % rapeseed oil were successfully prepared and stabilized with 0.5 wt % CNFs, both an enzymatically treated quality, and a quality prepared by TEMPO-mediated oxidation. Both the CNF qualities produced emulsions that were stable towards coalescence and creaming over one month of storage. In particular, the emulsion stabilized with CNF-T obtained a very high viscosity, which made it stable towards centrifugation. For emulsions containing NaCl or acetic acid, the CNF-E-stabilized emulsions showed some increase in droplet size, but a very good time stability. In contrast, the CNF-T-stabilized emulsions became highly unstable upon addition of NaCl or acid. This was also reflected in a large loss of viscosity, and points towards the great importance of the electrostatic repulsion between fibrils for the high viscosity and high stability of emulsions stabilized with highly charged CNFs. The results are promising enough to warrant further studies on CNF-E in food emulsions.

## Figures and Tables

**Table 1:** Added amount of acetic acid (AcOH) and NaCl to a series of 0.5 wt % CNFs and 40 wt % rapeseed oil emulsions.

Sample	NaCl	AcOH
	(wt %)	(wt %)
CNF-E	-	-
CNF-E + AcOH	-	0.2
CNF-E + NaCl	1.0	-
CNF-E + NaCl + AcOH	1.0	0.2
CNF-T	-	-
CNF-T + AcOH	-	0.2
CNF-T + NaCl	1.0	-
CNF-T + NaCl + AcOH	1.0	0.2

**Table 2:** Carboxyl group content based on conductometric titration and residual fiber content calculated using data from fiber analysis (L&W Fiber tester plus). The number of passes during microfluidization is presented in brackets for the two CNF samples.

	CNF-E (5 Passes)	CNF-T (1 Pass)
Total charge (mmol/g)	0.044 ± 0.003	1.49 ± 0.02
Residual fiber content (%)	0.1 ± 0.0	5.5 ± 0.2

**Table 3:** Apparent viscosity of CNF-stabilized emulsions in mPa∙s (average value and coefficient of variance (Cv) based on two replicates). The media are the following. Ref: deionized water; AcOH: 0.2 wt % acetic acid; NaCl: 1.0 wt % NaCl; AcOH + NaCl: 0.2 wt % acetic acid + 1.0 wt % NaCl.

CNF Sample	0.5 wt % CNF-E				0.5 wt % CNF-T			
Medium	Ref	AcOH	NaCl	AcOH + NaCl	Ref	AcOH	NaCl	AcOH + NaCl
Average viscosity in mPa∙s	5324	7277	2809	5217	38305	9122	1311	4013
Cv	5.5%	2.2%	2.8%	5.6%	0.6%	3.8%	35%	21%

**Table 4:** Measured pH for CNF dispersions and for CNF-stabilized emulsions. The media are the following. Ref: deionized water; AcOH: 0.2 wt % acetic acid; NaCl: 1.0 wt % NaCl; AcOH + NaCl: 0.2 wt % acetic acid + 1.0 wt % NaCl.

CNF Sample	0.5 wt % CNF-E					0.5 wt % CNF-T				
	Dispersion	Emulsions				Dispersion	Emulsions			
Medium	CNF dispersion	Ref	AcOH	NaCl	AcOH + NaCl	CNF dispersion	Ref	AcOH	NaCl	AcOH + NaCl
pH	7.52	7.31	3.22	5.55	3.02	6.91	6.04	3.95	6.42	3.42
